# Does Uptake of Pharmaceuticals Vary Across Earthworm Species?

**DOI:** 10.1007/s00128-016-1875-7

**Published:** 2016-07-21

**Authors:** Laura J. Carter, Jim J. Ryan, Alistair B. A. Boxall

**Affiliations:** 1 Environment Department, University of York, Heslington, York, YO10 5DD UK; 2 EHS Technical CoE, GlaxoSmithKline, Ware, SG12 0DP UK

**Keywords:** *Eisenia fetida*, *Lumbricus terrestris*, Species traits, Pharmaceutical, Uptake

## Abstract

This study compared the uptake and depuration of four commonly used pharmaceuticals (carbamazepine, diclofenac, fluoxetine and orlistat) in two earthworm species (*Lumbricus terrestris* and *Eisenia fetida*). *L. terrestris* are a larger species and often found in deep burrows whereas *E. fetida* prefer to reside near the soil surface. Species burrowing habits and sizes may alter uptake by earthworms. All four pharmaceuticals were taken up into both *L. terrestris* and *E. fetida* tissue after 21 days exposure to spiked soil. Bioconcentration factors (BCFs) ranged between 1.72 and 29.83 for *L. terrestris* and 1.14 and 63.03 for *E. fetida*. For carbamazepine and diclofenac, BCFs were similar whereas for fluoxetine and orlistat, BCFs in *E. fetida* were more than double those seen in *L. terrestris*. Results indicate that uptake into earthworms cannot be generalised between species and that the influence of species traits can vary depending on the nature of the study chemical.

Active pharmaceutical ingredients (APIs) can enter the soil environment when sludge from wastewater treatment plants is used as a fertiliser or when wastewater effluent is used for irrigation purposes (Golet et al. 2002; Kinney et al. 2006a, b). Following entry into soil systems, many APIs can persist and have been detected in soils at µg/kg concentrations (Dalkmann et al. 2012; Duran-Alvarez et al. 2009; Siemens et al. 2008) where there is the potential to be taken up by soil dwelling organisms, such as earthworms (Berge and Vulliet 2015; Carter et al. 2014b; Kinney et al. 2008).

In a recent study, we explored the uptake of a range of APIs, with different physico-chemical properties, into the epigeic earthworm, *Eisenia fetida*. The APIs were found to accumulate in *E. fetida* with pore water based bioconcentration factors ranging from 2.25 (carbamazepine) to 51.5 (orlistat) (Carter et al. 2014b). *Eisenia fetida* was selected in these studies as it is the preferred standard earthworm species in many international regulatory guidelines for risk assessment such as the Organisation of Economic and Cooperative Development Bioaccumulation in Terrestrial Oligochaetes test (OECD 317) (OECD 2010). However, a number of different earthworm species co-exist within the soil environment and studies with non-pharmaceutical contaminants (e.g. DDE and metals) indicate that chemical uptake and toxicity can vary across earthworm species (Kelsey et al. 2005; Langdon et al. 2005; Spurgeon and Hopkin 1996). In addition to the role of chemical properties and soil parameters, which are known to influence chemical bioavailability, these differences in uptake are thought to be due to differences in processing of soil organic matter, ecological strategy, and lipid content across the earthworm species studied (Kelsey et al. 2005). Based on these previous studies, it is likely that uptake of APIs will also differ across earthworm species. In order to adequately assess the risks of APIs in the terrestrial environment it would therefore be worthwhile to characterise these differences.

In this study, we therefore explore the uptake of four commonly used human pharmaceuticals (carbamazepine, diclofenac, fluoxetine and orlistat) (Table 1) into the earthworm, *Lumbricus terrestris* and compare and contrast the results with the findings from our previous work with *E. fetida.* The selected earthworm species have different characteristics or traits such as lipid content and size and prefer different temperature conditions and diets. These differences may influence the uptake and bioaccumulation of APIs (Table 2).

**Table 1:** Test chemical physico-chemical properties

API	Formula	Molar mass (g/mol)	Log *K*_*ow*_^a^	p*K*_a_^a^	Solubility (mg/mL)^a^	Sorption coefficient (*K*_*d*_) (L/kg)^b^	Specific activity (GBq/mmol)
Carbamazepine	C_15_H_12_N_2_O	236.30	2.67	14.3	0.084	4.83 ± 0.68	0.74
Diclofenac	C_14_H_11_Cl_2_NO_2_	318.13	4.06	4.4	0.016	28.7 ± 3.27	2.30
Fluoxetine	C_17_H_18_F_3_NO	345.80	4.09	9.6	0.032	608 ± 87.6	2.04
Orlistat	C_29_H_53_NO_5_	497.74	8.95	13.1	0.016	1494 ± 103	2.05

^a^Predicted from ACD/I-Labs

^b^Carter et al. (2014b)

**Table 2:** Earthworm characteristics of *Eisenia fetida* and *Lumbricus terrestris*

	*Eisenia fetida*	*Lumbricus terrestris*
Ecological grouping	Epigeic	Anecic
Time to maturity (days)	28–30^a^	112 at 15°C^c^
Colour	Brown and buff bands^a^	Brownish-purple/red above; yellow orange below^b^
Optimal temperature (^o^C)	25 (0–35)^a^	~10^d^
Length (mm)	60–120^b^	90–350^b^
Diameter (mm)	3–6^b^	6–10^b^
Number of segments	80–120^b^	140–155^b^
Mode of reproduction	Obligatory amphimictic	Obligatory amphimictic
Cocoon incubation time	18–26^a^	90 at 15°C^e^
Where in soil profile?	Leaf litter/surface^b^	Deep burrows^b^
Soil pH preference	4.3–7.5^b^	6.2–10.0^b^

^a^Domínguez (2004)

^b^Sims and Gerard (1999)

^c^Svendsen et al. (2002)

^d^Edwards and Bohlen (1996)

^e^Butt (1991)

## Materials and Methods

The test chemicals were ^14^C labelled compounds (Table 1). Fluoxetine and carbamazepine were obtained from American Radiolabelled Chemicals (Missouri, USA), diclofenac was obtained from Perkin Elmer (Boston, USA) and orlistat was kindly provided by GlaxoSmithKline (GSK, UK). Solvents including acetonitrile (99.9 %), methanol (99.9 %) and ethyl acetate (99.9 %) were HPLC grade and obtained from Fisher Scientific (Loughborough, UK). The test soil was a clay loam (soil 280) obtained from LandLook (Midlands, U.K.) and had been used in earlier earthworm uptake studies with *E. fetida* (Carter et al. 2014b). Prior to the uptake studies, the field fresh soil was air dried then sieved to 2 mm to ensure homogeneity within the soil matrix. Soil 280 had an organic matter content of 3 %, a pH (water) of 6.3, which can be tolerated by both species of earthworm and a total organic carbon concentration of 1.89 % which would ensure moderate bioavailability of the APIs.

*Lumbricus terrestris* were obtained from Blades Biological Ltd. (Kent, UK) and were cultured in a plastic box containing 8 kg of soil 280 and kept in a growth chamber under experimental conditions (see below) prior to use in the uptake studies. They were fed twice weekly with birch leaves and pre-treated horse manure which was dried at 105°C and then rewetted, both of which were applied to the top of the culture medium. The mean lipid content of *L. terrestris* has been reported in literature as 1.23 % ± 0.20 % based on fresh weight (Albro et al. 1992).

Experiments followed the minimised design approach described by Carter et al. (2014a). For each API, 12 adult *L. terrestris* (4.03 ± 0.87 g wet weight) were individually exposed to 350 g API spiked soil for 21 days (uptake phase) containing either 119.1, 76.8, 46.9 or 71.3 Bq/g of carbamazepine, diclofenac, fluoxetine or orlistat respectively. The equivalent parent compound concentrations were 36, 9.9, 7.2 and 15.8 µg/kg. Exposures were to individual APIs, not as a mixture, and the APIs prepared in a carrier solvent (methanol or acetonitrile), spiked directly into the soil (0.8–1.5 mL/beaker) and mixed by hand to achieve a homogeneous distribution of the chemical. After spiking beakers were left for 48 h to evaporate any solvent residues. Blank and solvent controls were also prepared (n = 6). The moisture content of the soils was monitored throughout the study and if necessary adjusted with deionised water to maintain the soil at 40–60 % of the maximum water holding capacity (MWHC). Beakers containing soil and earthworms were incubated under controlled conditions to a constant dark cycle at 13 ± 2°C and 60 % humidity and *L. terrestris* were fed twice weekly. Following the 21 days exposure, six *L. terrestris* were removed from each beaker for each API and left on moist filter paper for 30 h to purge their guts. The remaining earthworms were transferred to 350 g of clean soil for a further 21 days to explore the depuration of the APIs (six replicates per API). After depuration, the earthworms were removed and allowed to void their gut contents. Following collection, all *L. terrestris* were immediately frozen (−20°C) until analysis. Samples of soil were taken at the beginning and end of the exposures for total soil and soil pore water analysis.

Pore water, soil and earthworms were extracted using the methods outlined in Carter et al. (2014b). Pore water was extracted by centrifugation whilst soil and earthworm samples were extracted by solvent extraction. Combustion analysis of the soil was also performed to determine if there was radioactivity remaining in the soil in the form of non-extractable residues. Concentrations of the study pharmaceuticals in pore water, soil and earthworm extracts were determined using liquid scintillation counting on a Beckman LS 6500 LSC counter (Beckman Coulter Inc., Fullerton, USA). Each sample was counted three times for 5 min. Counts were corrected for background activity by using blank controls. Counting efficiency and colour quenching were corrected using the external standard ratio method.

Measured radioactivity in the pore water samples and tissues were input into Eqs. 1 and 2 to derive uptake (*k*1) and depuration rates (*k*2) for *L. terrestris* in each exposure. The minimised design approach assumes that chemical uptake follows first order principles, such that the rate of uptake and depuration are directly proportional to the concentration of the pharmaceutical in the tissue (Carter et al. 2014a).

1 $$k2 = \frac{{\ln {C}_{t1} {-} \ln {C}_{t2} }}{\text{t}_{\text{d}}}$$

2 $$k1 = \frac{{k2{{C}}_{{{{t}}1}} }}{{{{C}}_{{pw}} \left( {1 - {\text{e}}^{-{k}2{\text{t}}_{\text{u}} }} \right)}}$$

where *k*2 is the depuration rate constant, *k*1 is the uptake rate constant, *C*_*pw*_ is mean pore water concentration during exposure phase, *C*_*t*__1_ and *C*_*t*2_ are the average earthworm concentrations after the uptake and depuration phases respectively and t_u_ and t_d_ are the lengths of the uptake and depuration period respectively.

The uptake and depuration rates were then used to estimate pore water based kinetic bioconcentration factors (BCFs) (Eq. 3).

3 $$BCFpw = \frac{k1}{k2}$$

For comparison, data from previous full uptake and depuration *E. fetida* experiments (Carter et al. 2014b) was resampled according to if the experiment had been carried out using the minimised design principles to generate an equivalent minimised design pore water based BCFs to that calculated for *L. terrestris* (Table 3). Measured data used in these calculations was originally obtained from full uptake and depuration studies according to OECD 317 (OECD 2010).

**Table 3:** Kinetic parameters (*k*1 and *k*2) together with BCFs derived using the minimised design approach for *L. terrestris* and *E. fetida*

	*C*_*t*1_ (Bq/g) (internal)	*C*_*t*2_ (Bq/g) (internal)	Mean *C*_*pw*_ (Bq/mL)	*k*2 (dep. rate) (day^−1^)	*k*1 (uptake rate) (mL/g day^−1^)	Pore water BCF
*Lumbricus terrestris* (this study)						
Carbamazepine	20.88 ± 8.77	1.35 ± 0.22	13.02 ± 3.87	0.13	0.22	1.72
Diclofenac	8.38 ± 3.21	7.73 ± 2.76	3.62 ± 0.61	0.004	0.12	29.83
Fluoxetine	8.52 ± 3.49	1.59 ± 1.01	0.62 ± 0.39	0.08	1.35	16.87
Orlistat	7.29 ± 1.92	3.21 ± 1.06	1.09 ± 0.58	0.04	0.47	11.93
*Eisenia fetida* [raw data re-sampled from Carter et al. (2014b)]						
Carbamazepine	30.42 ± 5.83	0.37 ± 0.35	27.05 ± 4.90	0.21	0.24	1.14
Diclofenac	155.9 ± 26.6	40.61 ± 4.9	9.16 ± 1.95	0.06	1.48	23.03
Fluoxetine	137.38 ± 10.8	16.19 ± 12.1	3.56 ± 0.50	0.10	4.46	43.76
Orlistat	66.73 ± 10.89	39.14 ± 4.38	2.56 ± 0.57	0.03	1.60	63.03

Average (±standard deviation) measured earthworm tissue concentrations provided at the end of uptake phase (*C*_*t*1_) and depuration phase (*C*_*t*2_) and mean API concentration in the pore water during the uptake phase (*C*_*pw*_)

## Results and Discussion

**Fig. 1:**
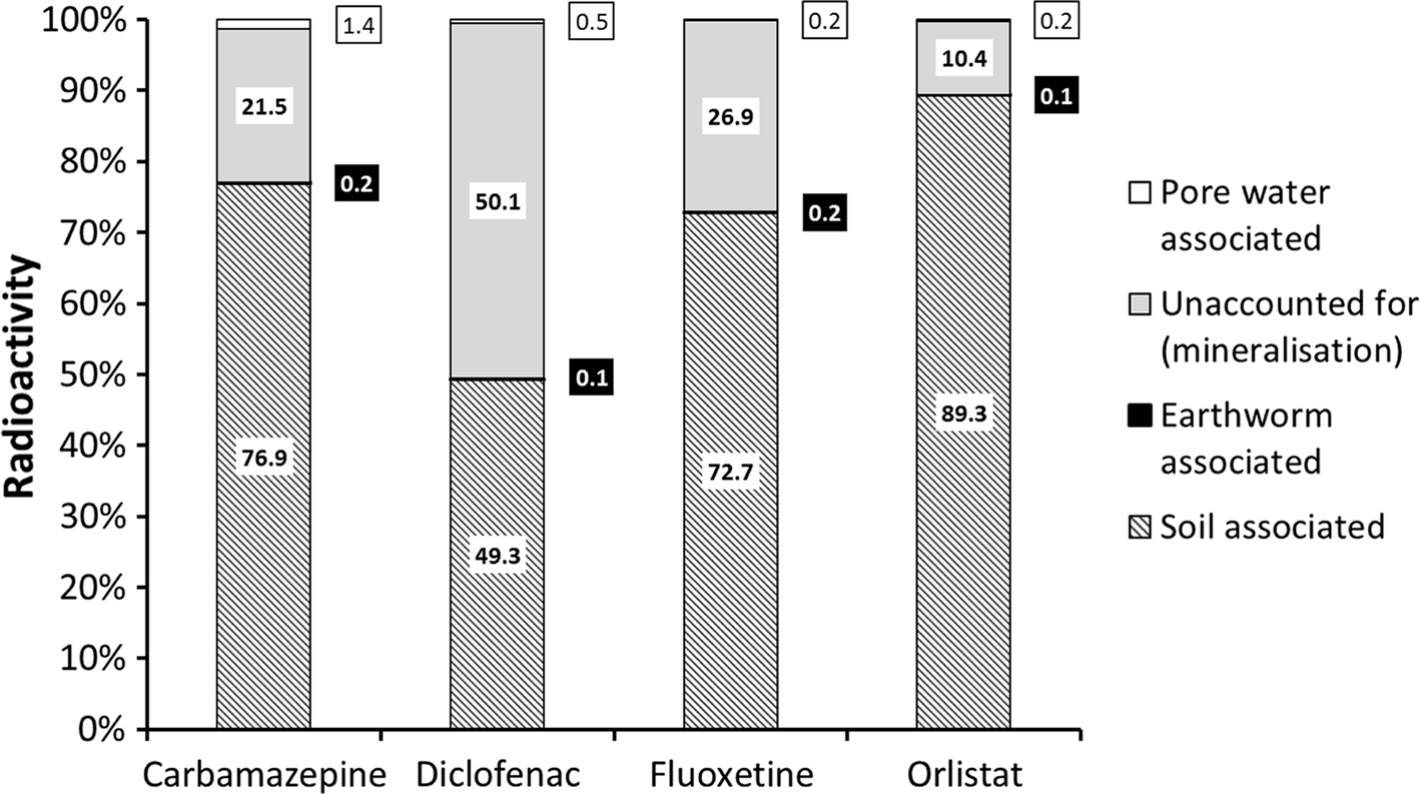
Mass balance describing radioactivity present in system after 21 days. Radioactivity associated with the soil (*diagonal*) earthworm (*black*) pore water (*white*) were calculated as a percentage of initial radioactivity applied (day 0) with radioactivity unaccounted for denoted by a *grey bar*

At the start of the experiment (0 days), for all treatments, the largest amount of the applied radioactivity was associated with the soil phase. The greatest proportion of the radioactivity associated with the pore water was measured in the carbamazepine treatment (1.24 % ± 0.10 %) whereas the smallest proportion in the pore water was seen for the fluoxetine treatment (0.18 % ± 0.06 %) suggesting that the fluoxetine was more strongly sorbed to the soil (Fig. 1). After the 21 days uptake phase, most of the radioactivity remained associated with the soil with 77 %–89 % of the applied radioactivity recovered from the soil via extraction for all treatments except diclofenac where only 49 % was accounted for (Fig. 1). For all treatments, a proportion of the applied radioactivity was unaccounted for after 21 days, potentially due to loss via mineralisation. Anti-inflammatory APIs are known to degrade rapidly whereas carbamazepine and fluoxetine are more persistent in a range of soil types (Monteiro and Boxall 2009).

A small amount of radioactivity was taken up and accumulated by *L.* terrestris. After 21 days exposure, 1.96 % ± 0.65 %, 1.17 % ± 0.57 %, 2.11 % ± 1.04 % and 0.72 % ± 0.18 % of the applied radioactivity was taken up by *L. terrestris* in the carbamazepine, diclofenac, fluoxetine and orlistat studies respectively. Uptake rates (*k*1) were greatest for fluoxetine followed by orlistat, carbamazepine and diclofenac (Table 3). Carbamazepine was found to be depurated fastest followed by fluoxetine, then orlistat and diclofenac. For diclofenac, approximately 90 % of the accumulated radioactivity remained in the *L. terrestris* after the depuration period. Over time, significant chemical uptake in combination with a slow depuration rate would present a greater risk of transfer from one trophic level to another than a fast depurating chemical. In general, the more hydrophobic chemicals were taken up fastest by *L. terrestris* however trends relating to depuration rates and hydrophobicity were not evident. Pore water based bioaccumulation factors (BCFs) increased in the order of diclofenac > fluoxetine > orlistat > carbamazepine for *L. terrestris* after exposure to the pharmaceuticals in soil 280 and ranged from 1.72 to 29.83 (Table 3). Unlike the BCFs we have seen previously for *E. fetida*, BCFs in *L. terrestris* did not increase with increasing hydrophobicity of the chemical which suggests that additional factors, such as species traits, are important in determining the degree of bioaccumulation of pharmaceuticals in the larger earthworm.

Comparison of the results for *L. terrestris* with results from our previous work on *E. fetida* shows that, with the exception of carbamazepine, uptake rates (*k*1) were faster in *E. fetida* in comparison to *L. terrestris*. The depuration rates for *L. terrestris* are similar to those seen for *E. fetida* (Table 3). The pore water based BCFs in *L. terrestris* for carbamazepine and diclofenac were larger than in *E. fetida*, however these differences were small at less than a factor of 2. Meanwhile BCFs for fluoxetine and orlistat were larger in the smaller species, *E. fetida* by a factor of 2.5 and 5, respectively (Table 3). While smaller BCFs calculated for fluoxetine and orlistat in the larger species *L. terrestris* follow a similar pattern to the findings of Kelsey et al. (2005), the observations for diclofenac and carbamazepine contradict this as for these APIs the larger BCFs were observed in the smaller species and indicate that the influence of species traits on uptake varies depending on the properties of the chemical.

The uptake of organic chemicals can be related to hydrophobicity and the lipid content of the organism as well as differences in the design of the exposures such as test conditions used, food source, temperature and lighting regime. While the food source in the two studies were different, it is unlikely that these differences explain the observations for the two species as studies with sediment dwelling worms have indicated that uptake of two of the study pharmaceuticals is via the pore water and not via ingestion (Karlsson et al. 2016). Even though the effect of chemical concentration on API uptake is yet to be explored in terrestrial invertebrates approximately similar BCFs have been reported in fish at low and high API concentrations (Lahti et al. 2011). Given that differences in exposure concentrations between the two test organisms in the current study were a maximum of an order of magnitude, concentration is unlikely to explain the differences in BCFs. Temperature differences may explain the differences as increased temperatures will increase metabolism and degradation in the soil matrix (Monteiro and Boxall 2009). In the fluoxetine and orlistat exposures, differences in lipid content between the two earthworm species may be responsible for the higher BCFs in *E. fetida* as this species has higher lipid content (5.1 % wet weight) (Carter et al. 2014b). Combined with the fact that both these chemicals are hydrophobic, orlistat in particular with a log K_ow_ value of 8.95 (Table 1), this would infer a higher propensity for uptake into lipids and thus may account for the larger *E. fetida* BCF. Furthermore, based on the idea that as the size of an object increases the surface area to volume ratio decreases, *E. fetida* being the smaller earthworm species would have greater potential for the diffusion of chemicals through their tissues than the larger, *L. terrestris*. Previous research elucidating the uptake of chemicals into earthworms demonstrated that as the hydrophobicity of the chemicals uptake via the gut route became increasingly important (Belfroid et al. 1995; Jager et al. 2003). A combination of large surface area to volume ratio ensuring minimal uptake via diffusion and the hydrophobic nature of orlistat restricting uptake to primarily across the gut wall may explain the smaller BCFs generated in this study for *L. terrestris* in comparison to *E. fetida*.

However, for carbamazepine and diclofenac exposures, the results presented in this study do not follow these established trends as a larger BCF was observed for *L. terrestris.* Additional mechanisms or processes must exist for carbamazepine and diclofenac which result in higher BCFs for *L. terrestris*. As these APIs are more hydrophilic than fluoxetine and orlistat, diffusion across the earthworm skin is the dominant route of exposure (Jager et al. 2003) (Table 1) therefore the larger volume of tissue in the *L. terrestris* could facilitate a higher capacity for uptake, shown by faster uptake rates (*k*1), or greater storage ability of the chemicals in the tissue, as shown by the larger proportion of radioactivity measured in the earthworm (*C*_*t*1_*)* at the end of the uptake phase (Table 3).

The earthworms considered in this analysis have contrasting behaviour in the soil environment. *Eisenia fetida* is an epigeic species which primarily lives at or near the soil surface and consumes coarse particulate organic matter and surface litter whilst anecic species, such as *L. terrestris* live in deep burrows and come to surface to feed on surface litter (Bouché 1983). As the test chemicals were spiked and thoroughly mixed in the soil to create an even distribution of the APIs, the differences in earthworm ecology between *L. terrestris* and *E. fetida* are probably not responsible for the differences in BCFs between species. However, this heterogeneity is not necessarily representative of the natural soil environment where APIs will most likely be applied to the top layers of the soil profile after addition of biosolids or use of reclaimed wastewater irrigation (Gibson et al. 2010). In the natural environment, earthworms which prefer to reside near the soil surface (*endogeic* species) would therefore have a greater exposure to chemicals than the deep burrowing species, which come to the surface less often (*anecic* species). Hydrophilic pharmaceuticals, which have a greater potential for movement with percolating water flows, may be more widely distributed in the soil profile than highly sorptive pharmaceuticals which are retained near the soil surface (Table 1). This may contribute to a greater uptake of weakly sorbed pharmaceuticals in species such as *L. terrestris* which have deep vertical burrows stretching 1–2 m (Sims and Gerard 1999). In comparison, chemicals with a particularly hydrophobic nature in a soil with high organic content will not be leached easily and thus remain near the soil surface where uptake by upper soil species may occur in preference to subsoil dwellers.

The BCFs reported in this study are all relatively small (<100) and would suggest the potential for food chain transfer and secondary toxicity is minimal. Nevertheless, differences in pharmaceutical physico-chemical properties (Table 1) in combination with species habitat preference and soil parameters can affect earthworm uptake in the natural environment. Additional studies are required to determine whether this needs to be addressed with regards to risk assessment using a broader range of soil types and pharmaceutical compounds. It is important to recognise that only two species were evaluated in this study and therefore to draw more specific conclusions it may be necessary to look at a wider range of earthworm species for example with differing burrowing habits, soil property preferences and sizes. Recent research has demonstrated that different benthic invertebrate species with distinct bioturbation modes can affect the direction and magnitude of the remobilisation of organic contaminants bound to sediments (Bundschuh et al. 2016). More work is required to elucidate how species characteristics can affect the fate and uptake of chemicals in soil systems. In addition to oligochaetes there are a number of other invertebrates in the terrestrial environment, such as springtails, insects and snails. If APIs are present in the soil, all of these species will be exposed, not just earthworms. Further research is therefore required to evaluate the effect of species traits (e.g. if an exoskeleton changes API uptake) using a wider variety of test organisms.
